# Subthalamic high-beta oscillation informs the outcome of deep brain stimulation in patients with Parkinson's disease

**DOI:** 10.3389/fnhum.2022.958521

**Published:** 2022-09-08

**Authors:** Po-Lin Chen, Yi-Chieh Chen, Po-Hsun Tu, Tzu-Chi Liu, Min-Chi Chen, Hau-Tieng Wu, Mun-Chun Yeap, Chih-Hua Yeh, Chin-Song Lu, Chiung-Chu Chen

**Affiliations:** ^1^Division of Movement Disorders, Department of Neurology, Chang Gung Memorial Hospital, Taoyuan, Taiwan; ^2^Neuroscience Research Center, Chang Gung Memorial Hospital, Taoyuan, Taiwan; ^3^College of Medicine, Chang Gung University, Taoyuan, Taiwan; ^4^Department of Neurosurgery, Chang Gung Memorial Hospital, Taoyuan, Taiwan; ^5^Department of Mathematics, National Taiwan University, Taipei, Taiwan; ^6^Department of Public Health, Biostatistics Consulting Center, College of Medicine, Chang Gung University, Taoyuan, Taiwan; ^7^Department of Obstetrics and Gynecology, Chang Gung Memorial Hospital, Chiayi, Taiwan; ^8^Department of Mathematics, Duke University, Durham, NC, United States; ^9^Department of Statistical Science, Duke University, Durham, NC, United States; ^10^Department of Neuroradiology, Chang Gung Memorial Hospital, Taoyuan, Taiwan; ^11^Professor Lu Neurological Clinic, Taoyuan, Taiwan

**Keywords:** deep brain stimulation, Parkinson's disease, beta oscillations, subthalamic nucleus (STN), stimulation efficacy

## Abstract

**Background:**

The therapeutic effect of deep brain stimulation (DBS) of the subthalamic nucleus (STN) for Parkinson's disease (PD) is related to the modulation of pathological neural activities, particularly the synchronization in the *β* band (13–35 Hz). However, whether the local *β* activity in the STN region can directly predict the stimulation outcome remains unclear.

**Objective:**

We tested the hypothesis that low-*β* (13–20 Hz) and/or high-*β* (20–35 Hz) band activities recorded from the STN region can predict DBS efficacy.

**Methods:**

Local field potentials (LFPs) were recorded in 26 patients undergoing deep brain stimulation surgery in the subthalamic nucleus area. Recordings were made after the implantation of the DBS electrode prior to its connection to a stimulator. The maximum normalized powers in the theta (4–7 Hz), alpha (7–13 Hz), low-*β* (13–20 Hz), high-*β* (20–35 Hz), and low-γ (40–55 Hz) subbands in the postoperatively recorded LFP were correlated with the stimulation-induced improvement in contralateral tremor or bradykinesia–rigidity. The distance between the contact selected for stimulation and the contact with the maximum subband power was correlated with the stimulation efficacy. Following the identification of the potential predictors by the significant correlations, a multiple regression analysis was performed to evaluate their effect on the outcome.

**Results:**

The maximum high-*β* power was positively correlated with bradykinesia–rigidity improvement (*r*_*s*_ = 0.549, *p* < 0.0001). The distance to the contact with maximum high-*β* power was negatively correlated with bradykinesia–rigidity improvement (*r*_*s*_ = −0.452, *p* < 0.001). No significant correlation was observed with low-*β* power. The maximum high-*β* power and the distance to the contact with maximum high-*β* power were both significant predictors for bradykinesia–rigidity improvement in the multiple regression analysis, explaining 37.4% of the variance altogether. Tremor improvement was not significantly correlated with any frequency.

**Conclusion:**

High-*β* oscillations, but not low-*β* oscillations, recorded from the STN region with the DBS lead can inform stimulation-induced improvement in contralateral bradykinesia–rigidity in patients with PD. High-*β* oscillations can help refine electrode targeting and inform contact selection for DBS therapy.

## Introduction

High-frequency stimulation directed at the subthalamic nucleus (STN) is an effective therapy for patients with Parkinson's disease (PD) (Krack et al., 2003; Weaver et al., 2009; Follett et al., 2010). The selection of the active stimulation contact in postoperative programming is typically based on empirical trials by experienced neurologists (Chen et al., 2003; Volkmann et al., 2006). With the advancements in imaging and connectomics, enhanced therapeutic benefits have been achieved through the selection of the optimal contact location (Vanegas-Arroyave et al., 2016; Akram et al., 2017; Horn et al., 2017b; Dembek et al., 2019; Boutet et al., 2021). However, whether the local field potentials (LFPs) in the STN recorded during the perioperative period predict the outcome of deep brain stimulation (DBS) remains inconclusive (Ray et al., 2008; Zaidel et al., 2010; Boex et al., 2018).

The efficacy of STN DBS may rely on the modulation of the pathological neural activity in the STN area (Zaidel et al., 2010; Accolla et al., 2016; Horn et al., 2017a; Milosevic et al., 2020; Kehnemouyi et al., 2021). Exaggerated *β* (13–35 Hz) oscillations in the “motor domain” of the cortical–subcortical network have been suggested as the key biomarker of motor impairments in patients with PD (Brown, 2003; Hammond et al., 2007; Eusebio et al., 2009; Little and Brown, 2014). Suppression of such *β* activity by levodopa or DBS is associated with the improvement of parkinsonism (Brown et al., 2001; Levy et al., 2002; Priori et al., 2004; Weinberger et al., 2006; Ray et al., 2008; Bronte-Stewart et al., 2009; Kühn et al., 2009; Eusebio et al., 2011). Electrophysiological and volume of tissue-activated modeling studies have indicated that the proximity of the chronic stimulation contact to the sensorimotor STN *β* oscillations predicts a favorable therapeutic outcome with STN DBS (Butson et al., 2007; Horn et al., 2017a).

Previous studies have also suggested that there were different pathophysiology and clinical relevance between low-*β* (13–20 Hz) and high-*β* frequency (20–35 Hz) activities in the STN (Priori et al., 2004; Marceglia et al., 2006; Oswal et al., 2016; Godinho et al., 2021; Neuville et al., 2021). However, whether and how the subthalamic *β* oscillations at different subbands predict the therapeutic outcome of DBS in PD remains unclear.

In the current study, we investigated the association between the therapeutic efficacy of STN DBS and the LFPs recorded by the implanted DBS electrode during the perioperative period. First, different frequency bands were correlated with the stimulation efficacy to evaluate whether there was a frequency-specific association. Second, we explored whether the proximity of the active contact to the maximum *β* oscillations was also correlated with the stimulation efficacy. Lastly, a multiple regression analysis was performed to evaluate the combined predictive value of the factors that were significantly associated with the stimulation efficacy.

## Materials and methods

### Patients and surgery

Twenty-six patients with advanced PD who underwent bilateral STN DBS from 2009 to 2016 at Chang Gung Memorial Hospital (CGMH), Taiwan, were recruited (nine women; age, 61.0 ± 7.6 years; disease duration, 14.4 ± 5.8 years; preoperative baseline OFF-medication Unified Parkinson's Disease Rating Scale [UPDRS] III score, 45.0 ± 15.0). The Ethics Review Board in CGMH approved the study (CGMH-IRB No. 97-2345B), and informed consent was obtained from all patients. The patients' clinical characteristics are summarized in Table 1.

**Table 1:** Patient characteristics.

**No**.	**Sex**	**Age (y)**	**Disease duration (y)**	**Pre-op UPDRS III ON/OFF MED**	**Pre-op LEDD**	**Motor subtype**	**Main disabling symptoms**	**Chronic stimulation contacts^a^**
1	F	60	10	19/37	1,358	AR	Motor fluctuation	C3, C10
2	M	71	11	22.5/38.5	1,836	AR	Motor fluctuation, Dyskinesia	C2, C10
3	F	55	14	9/48.5	1,524	T	Motor fluctuation, Dyskinesia	C3, C11
4	F	70	6	21/42	900	AR	Motor fluctuation	C1, C10
5	M	56	16	17/44	1,214	T	Motor fluctuation	C1, C11
6	F	60	23	24.5/27.5	1,248	T	Motor fluctuation	C3, C11
7	M	47	30	16/29	980	AR	Motor fluctuation, Dyskinesia	C3, C10
8	M	65	18	34/38	1,400	AR	Motor fluctuation, Dyskinesia	C2, C9
9	M	71	12	15/27	832	T	Motor fluctuation, Dyskinesia	C3, C11
10	F	69	11	28/61	1,364	AR	Motor fluctuation	C2, C11
11	M	48	5	47/74	1,836	AR	Truncal dystonia	C1, C10
12	M	53	13	20/31	1,820	AR	Axial rigidity, Dyskinesia	C3, C10
13	M	65	24	19.5/31.5	1,696	AR	Motor fluctuation, FOG	C2, C11
14	F	69	12	23/41	850	AR	Motor fluctuation	C2, C11
15	M	49	15	23/48	1,530	T	Dyskinesia	C3, C11
16	F	68	7	34/76	1,200	T	Motor fluctuation, Dyskinesia	C1, C9
17	M	70	16	13/29	1,150	AR	Motor fluctuation, FOG, VH to DA	C3, C11
18	M	57	9	23/47	799	AR	Motor fluctuation	C2, C9
19	F	54	17	18/34.5	1,790	AR	Motor fluctuation, Dyskinesia	C2, C8
20	M	53	24	28/44	710	AR	Biphasic dyskinesia	C0, C10
21	M	68	15	73/78	3,502.5	AR	Motor fluctuation, Dyskinesia	C3, C10
22	M	60	17	37/40	940	AR	Axial rigidity, FOG	C1, C9
23	M	56	13	26/55	892	T	Motor fluctuation, Axial rigidity	C3, C11
24	F	71	8	16/30	1,100	AR	Motor fluctuation, FOG	C3, C10
25	M	62	14	27/50	948	AR	Motor fluctuation	C3, C10
26	M	58	14	22/67.5	1,750	AR	Motor fluctuation	C1, C11

a Lead contacts C0–3 are on the left; C8–C11 are on the right.

F, female; M, male; T, tremor; AR, akinesia-rigidity; FOG, freezing of gait; VH, visual hallucinations; DA, dopamine agonist; UPDRS, Unified Parkinson's Disease Rating Scale; LEDD, levodopa equivalent daily dose.

The surgical procedures have been described in detail elsewhere (Chen et al., 2010, 2021). Preoperative magnetic resonance imaging (MRI) was done for the 26 patients, with 5 of them having high-resolution 1.5 tesla MRI records. Whole-brain stereotactic unenhanced computed tomography was obtained after the application of the Cosman-Roberts-Wells frame (Integra Radionics, Burlington, MA, USA) with a slice thickness of 1 mm. The images were transferred to the StealthStation S7 navigation system (Medtronic, Minneapolis, MN, USA) and superimposed to define the location corresponding to the STN in the atlas of Schaltenbrand and Wahren (Schaltenbrand and Wahren, 1977). The trajectories were then aimed at the center of STN under direct visualization on T2-weighted axial, coronal, and sagittal MRI.

To aid targeting, microelectrode recording (MER) was performed by an experienced neurophysiologist (C.C.C.) for all patients to ensure that the trajectories passed through the STN and to confirm the entry and exit levels of the STN, as well as the level of substantia nigra reticulata (SNr), based on the recognition of the typical transition of multi-unit firing patterns. The DBS lead (model 3389, Medtronic) was then implanted, with the lower border of contact C0 aimed at the level of the lower border of the STN or SNr, so that the contact C2 or C3 would lie in the sensorimotor domain of the STN. The correct placement of DBS leads in the region of the STN was verified by: (1) effective intraoperative macrostimulation, (2) postoperative T2-weighted MRI compatible with the placement of at least one lead contact in the STN region, and (3) when assessed 6 months postoperatively, a significant improvement in the UPDRS motor score during chronic DBS off medication compared to UPDRS off medication with stimulator switched off (44.3 ± 10.9 %, mean reduction ± SD; *p* < 10^−14^ with paired *t-*test).

### LFP recordings and signal processing

Resting-state LFPs were recorded within 5 days postoperatively, before the externalized leads were connected to the DBS pulse generator. Unipolar LFPs were recorded for ~200 s (213.0 ± 20.7 s) at a sampling rate of 2048 Hz *via* the TMSi-Porti amplifier (Twente Medical Systems International, Oldenzaal, Netherlands), using a common average reference as the built-in montage. LFPs were loaded onto a computer using custom software. For offline analysis, the LFP data were converted to an analyzable format using Spike2 software (Cambridge Electronics Design, Cambridge, UK).

The unipolar LFPs were analyzed using MATLAB 2019b (MathWorks, Natick, MA, Massachusetts). All LFPs were visually examined, and segments with artifacts were rejected. Two STNs (the right STN of patient 11 and the left STN of patient 25) were excluded due to poor recording quality, and the remaining 50 STNs were selected for further analysis. Locally weighted scatterplot smoothing with a span of 512 data points (0.25 s) was used for detrending. Spectral powers in different frequency bands (θ, 4–7 Hz; α, 7–13 Hz; low-*β*, 13–20 Hz; high-*β*, 20–35 Hz; and low-γ, 40–55 Hz) were calculated using a discrete Fourier transform. All powers were then normalized to the total power of 5–55 and 65–95 Hz frequency bands, avoiding contamination by movement artifacts (<5 Hz) and the power mains artifact (60 Hz in Taiwan). The maximum normalized power of each frequency band among four contacts was selected for further analysis of the correlation with the stimulation outcomes and with the clinical variables.

### Clinical evaluation

Initial programming of the stimulation parameters was done at 1 month postoperatively. During the initial programming session, a systematic evaluation of the contacts was performed. The stimulation was usually set at a frequency of 130 Hz and a pulse width of 60 μs, and the voltage was progressively increased at each contact to evaluate the stimulation effect on contralateral rigidity, bradykinesia, and tremor, as well as side effects. The contact that achieved the greatest improvement of motor symptoms with the least side effects was selected for chronic stimulation. The programming process was blind to any electrophysiological results. The stimulation intensity and dopaminergic medicine were adjusted gradually to avoid the occurrence of dyskinesia during the follow-up stage.

For clinical assessment, UPDRS-III ON/OFF stimulation after overnight withdrawal of dopaminergic medication was evaluated at about 1 year (0.8 ± 0.3 years, mean ± SD), with all subitems registered. The UPDRS hemibody scores were separated into the tremor (items 20 and 21), the bradykinesia–rigidity (items 22–26), and the axial (items 27–31) scores. The stimulation efficacy used in the main analysis was defined as follows:


$$\begin{array}{l} \text{Stimulation efficacy}=100\text{\%}\times \frac{\text{UPDR}{\text{S}}_{\text{OFF}-\text{MED},\text{ OFF}-\text{DBS}}-\text{UPDR}{\text{S}}_{\text{OFF}-\text{MED},\text{ ON}-\text{DBS}}}{\text{UPDR}{\text{S}}_{\text{OFF}-\text{MED},\text{ OFF}-\text{DBS}}} \end{array}$$

### Statistical analysis

Statistical analysis was performed using SPSS for Windows version 25 (IBM, Chicago, IL, USA). Due to the presence of extreme values, nonparametric Spearman's correlation was used for assessing the correlations between normalized power and stimulation efficacy, as well as for the correlation between distance and stimulation efficacy. Paired *t-*test was used to compare the maximum power locations in different frequency bands relative to the stimulation contacts. Spearman's correlation was used to explore the potential relationship between the baseline clinical variables (age, sex, disease duration, presence of levodopa-induced dyskinesia, daily levodopa equivalent dose, UPDRS-III during ON/OFF medication, and levodopa response) and the stimulation efficacy or the *β* powers; the Mann–Whitney *U-*test was used if dichotomized groups were compared (sex and presence of levodopa-induced dyskinesia). Lastly, a multiple linear regression model was computed to estimate the combined effects of the variables that were significantly correlated with the stimulation efficacy. Significance was indicated by *p* < 0.05.

## Results

**Figure 1:**
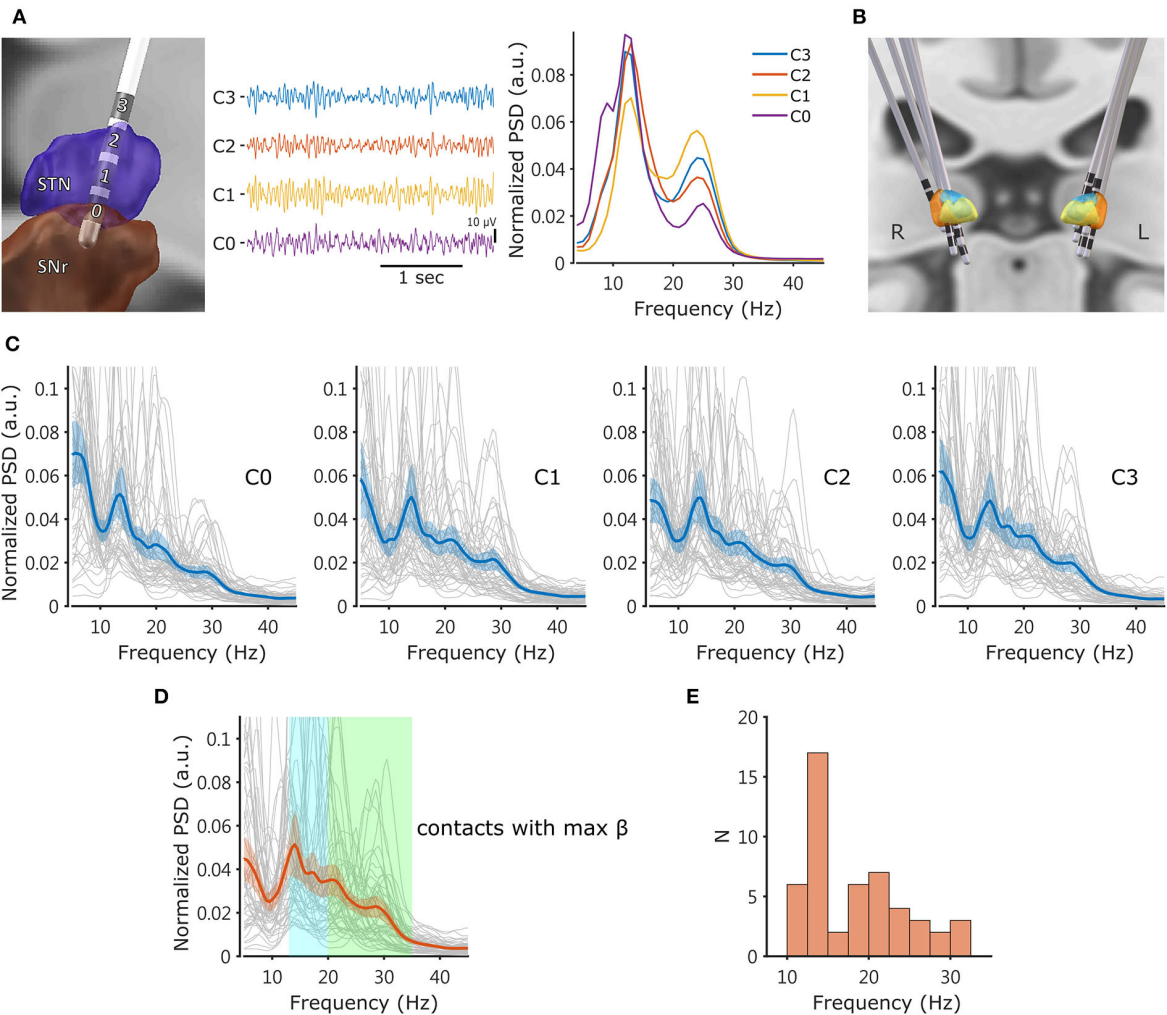
Example of DBS lead location and LFP. **(A)** Left: Lead-DBS reconstruction of an example lead located on the left STN of patient 14. Middle: The common average referenced unipolar LFPs from the same macroelectrode. Right: The normalized power spectra derived from the LFPs. Spectral peaks were observed at ~13 Hz and 24 Hz. The power spectra were generated using Welch's method and normalized so that the Y scale represents the spectral power relative to the total power (5–55 Hz and 65–95 Hz) in each 1-Hz bin. **(B)** Lead-DBS reconstruction of five patients' DBS leads, revealing that the electrodes were appropriately located at the sensorimotor STN. **(C)** Power spectra from every contact of all analyzed electrodes (*N* = 50 sides). Each thin line is an individual power spectrum. The thick solid line in each plot represents the averaged PSD, and the colored shadow indicates the 95% confidence interval of mean. **(D)** Power spectra from the contacts with the maximum normalized broad-band *β* power (13–35 Hz). The transparent rectangles in cyan and green mark the low-*β* (13–20 Hz) and high-*β* (20–35 Hz) frequencies, respectively. **(E)** Distribution of the peak frequencies (the local maximum within the 10–35 Hz range) of the power spectra from the contacts with the maximum broad-band *β*. LFP, local field potential; STN, subthalamic nucleus; SNr, substantia nigra pars reticulata; PSD, power spectral density.

Figure 1A illustrates an example of raw unipolar LFPs and power spectra from four contacts of the left STN in patient 14. The power spectra indicate a clear increase in power in the *β* frequency range (13–35 Hz). In this example of STN, two subpeaks centered at ~13 Hz and 24 Hz are evident. Figure 1B shows the reconstruction of the lead locations in five patients, and Figure 1C displays all power spectra from every contact of all patients. Figures 1D,E show the PSDs and the peak frequency distribution of the contacts with the maximum broad-band *β* power, respectively.

### Correlation between DBS efficacy and *β* oscillations

We explored whether the maximum oscillatory power in different frequency bands might be associated with the improvement of motor impairments due to STN DBS in PD. We correlated the maximum LFP power among four lead contacts at five frequencies and the improvement of motor impairments in response to DBS. The results of the correlation analysis are summarized in Table 2.

**Table 2:** Correlations between oscillation frequency and stimulation efficacy^a^.

**UPDRS improvement**	**Normalized power**				
	**θ**	**α**	**low-*β***	**high-*β***	**low-γ**
	**(4–7 Hz)**	**(7–13 Hz)**	**(13–20 Hz)**	**(20–35 Hz)**	**(40–55 Hz)**
Bradykinesia + rigidity	−0.039	−0.131	−0.055	0.549^***^	−0.110
(*n =* 50)	*p* = 0.786	*p* = 0.366	*p* = 0.704	*p* < 0.0001	*p* = 0.446
Tremor	0.100	0.069	−0.302	0.150	−0.109
(*n =* 39)	*p* = 0.544	*p* = 0.896	*p* = 0.061	*p* = 0.362	*p* = 0.507
Axial^b^	0.128	0.135	−0.246	0.026	0.289
(*n =* 26)	*p* = 0.534	*p* = 0.510	*p* = 0.226	*p* = 0.900	*p* = 0.152

a Spearman's correlation coefficients between the postoperative local field potential (LFP) powers and the improvement in contralateral motor symptoms with STN stimulation.

b Average power over bilateral STN was used for the correlation with the axial scores.

*** p < 0.001.

UPDRS, Unified Parkinson's Disease Rating Scale.

The maximum LFP in the high-*β* frequency range was positively correlated with the stimulation-induced improvement in contralateral bradykinesia–rigidity (*r*_*s*_ = 0.549, *p* < 0.0001). This correlation was frequency-specific, as no such association was noted between stimulation-related improvement in bradykinesia–rigidity and the maximum power in the theta (*r*_*s*_ = −0.039, *p* = 0.786), alpha (*r*_*s*_ = −0.131, *p* = 0.366), low-*β* (*r*_*s*_ = −0.055, *p* = 0.704), or low-gamma frequency range (*r*_*s*_ = −0.110, *p* = 0.446). In other words, the higher the high-*β* LFP power, the greater the improvement in contralateral bradykinesia–rigidity with stimulation (*R*^2^ = 0.267 with simple linear regression). However, improvement in the tremor on contralateral limbs was not significantly correlated with the maximum LFP power in any frequency range. The improvement in axial symptoms was not correlated with the LFP power in any frequency.

### Correlation between DBS efficacy and the distance between the contact with the maximum *β* oscillations and that used for chronic stimulation

**Figure 2:**
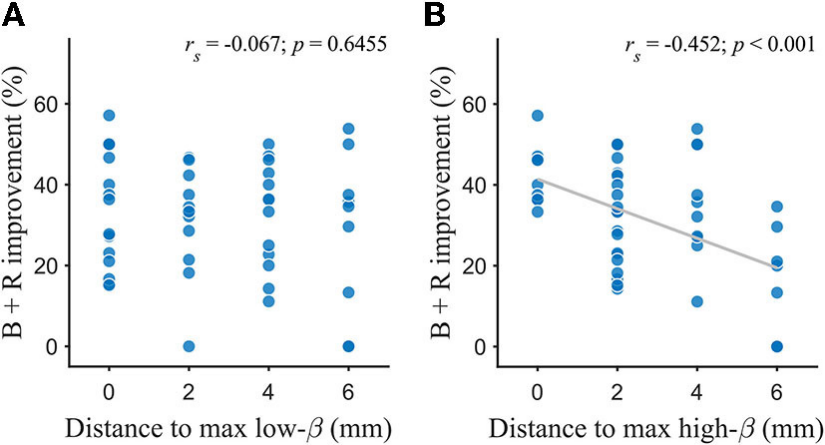
Association between the DBS efficacy and the distance between the contact with the maximum oscillations and the active contact. **(A)** Bradykinesia-rigidity improvement was not correlated with distance to max low-*β*. **(B)** Bradykinesia-rigidity improvement was negatively correlated with distance to max high-*β*. The distance was measured center-to-center between contacts. The fitted line was used to mark a trend. B, bradykinesia; R, rigidity; *r*_*s*_, Spearman's correlation coefficient.

Figure 2 shows the relationship between the distance to the maximum low-/high-*β* and the DBS efficacy for bradykinesia–rigidity. The DBS efficacy had a negative correlation with the center-to-center distance between the chronic stimulation contact and the contact with the maximum high-*β* power (*r*_*s*_ = −0.452, *p* < 0.001; Figure 2A). Stimulation at contact with the maximum high-*β* power was associated with greater therapeutic efficacy. This effect was frequency-specific because the distance to the contact with the maximum low-*β* power did not affect the stimulation outcome (*r*_*s*_ = −0.067, *p* = 0.645; Figure 2B).

The depths of the contacts with the maximum low-*β* and high-*β* power were compared. No significant difference was observed between the average depth of maximum low-*β* and maximum high-*β* (*p* = 0.830 with paired *t-*test).

### Results of the multiple regression of the high-*β* variables on the DBS efficacy

**Figure 3:**
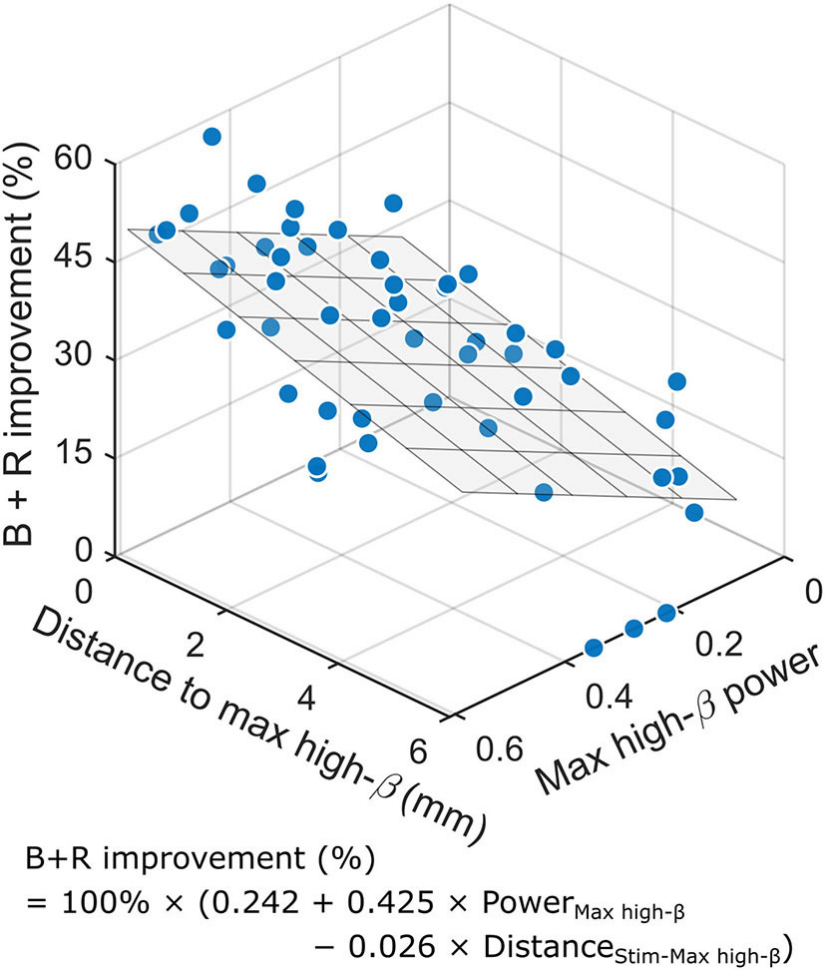
The maximum high-*β* power in the STN and the distance between the contact with maximum high-*β* oscillation and the active contact are significant predictors for the improvement in bradykinesia and rigidity with DBS. The 3D graph demonstrates the results of the multiple linear regression model. The higher the high-*β* power and the shorter the distance between the active contact and the contact with the maximum high-*β* oscillations, the greater the improvement in B + R due to DBS on contralateral limbs. The fitting formula is inset. The overall regression is significant [*R*^2^ = 0.374, *F*_(2,47)_ = 14.061, *p* =1.6 × 10^−5^]. The regression plane is displayed. B, bradykinesia; R, rigidity.

In the aforementioned correlation analyses, only two variables were identified to be significantly correlated with the stimulation efficacy on bradykinesia–rigidity, namely, the maximum high-*β* power and the distance to the maximum high-*β*. Thus, the two variables were put into a multiple linear regression as predictors to examine their combined effects on the contralateral bradykinesia–rigidity outcome. However, a moderate correlation between the two factors was found (*r*_*s*_ = −0.395, *p* = 0.005). To examine whether this collinearity is problematic, we performed a partial correlation analysis, which showed that both variables were still significantly correlated with the outcome when the effect of the other factor was partialized out (data not shown), and therefore they were adequately independent to each other in the current model. The regression model is shown in Figure 3. In this regression model, the slope coefficient for the maximum high-*β* power was 0.425 (95% CI: [0.139, 0.711], *p* = 0.0045), and the coefficient for the distance effect was −0.026 (95% CI: [−0.044, −0.008], *p* = 0.0058), which were both significant. The overall regression model was significant [*R*^2^ = 0.374, *F*_(2,47)_ = 14.061, *p* = 1.6 × 10^−5^]. Together, the power of the maximum high-*β* and the distance to the maximum high-*β* accounted for 37.4% of the variance in the therapeutic outcome in contralateral bradykinesia–rigidity.

### Relationship between low-/high-*β* oscillations and clinical characteristics

In order to identify a possible association between the baseline clinical characteristics and *β* oscillations, we correlated the patient's age, disease duration, motor scores, and levodopa responsiveness with maximum LFP power in the low-*β* and high-*β* frequency ranges. No significant association was found between the power in the two *β* frequency ranges and any of the clinical features (Table 3). The normalized low-*β* and high-*β* power were not correlated with the bradykinesia–rigidity score assessed postoperatively in the stimulation-off and medication-off state (*r*_*s*_ = −0.228, *p* = 0.112, and *r*_*s*_ = −0.025, *p* = 0.864, respectively). The occurrence of levodopa-induced dyskinesia (18 of 26 patients) was not associated with DBS outcome or LFP power in either *β* band (Table 3).

**Table 3:** Association of clinical characteristics with DBS efficacy and *β* frequency power.

	**Stim efficacy: B** + **R**		**Max LB power**		**Max HB power**	
	***r_*s*_***	***p***	***r_*s*_***	***p***	***r_*s*_***	***p***
**Pre-operative**
Age^a^	−0.027	0.895	0.216	0.311	0.074	0.731
Sex^a,b^	–	0.627	–	0.676	–	0.858
Disease duration^a^	0.055	0.789	−0.189	0.375	0.128	0.551
Dyskinesia^a,b^	–	0.397	–	0.378	–	0.974
LEDD^a^	0.122	0.553	0.441^*^	0.031	0.000	1.000
OFF UPDRS-III^a^	−0.025	0.904	0.181	0.396	0.010	0.961
ON UPDRS-III^a^	−0.078	0.705	0.283	0.181	−0.182	0.396
Levodopa response: UPDRS-III^a^	−0.047	0.821	−0.164	0.443	0.220	0.302
OFF UPDRS: B + R	−0.228	0.104	0.180	0.210	0.026	0.856
ON UPDRS: B + R	−0.161	0.255	0.230	0.109	−0.128	0.375
Levodopa response: B + R	−0.100	0.482	−0.087	0.550	0.119	0.412
**Post-operative**
OFF UPDRS: B + R	−0.162	0.252	−0.228	0.112	−0.025	0.864

a Average power over bilateral STN was used for the correlation with person-bound variables: age, sex, duration, dyskinesia, LEDD, and UPDRS-III.

^b^Mann–Whitney U-test was used for binary comparisons.

* p < 0.05.

LB, low-*β*; HB, high-*β*; B, bradykinesia; R, rigidity; UPDRS, Unified Parkinson's Disease Rating Scale; LEDD, levodopa equivalent daily dose; r_s_, Spearman's correlation coefficient.

## Discussion

We demonstrated that the high-*β* oscillations, as revealed by using subthalamic LFPs, helped in the estimation of the outcome of DBS therapy in patients with PD. Higher high-*β* power in the STN region and the proximity of the active contact to the maximum high-*β* oscillations were associated with a greater response to the DBS of the STN. A multiple linear regression model indicated that the spectral power at the high-*β* frequency band and the distance between the depth of the contact with the maximum high-*β* power and the active contact accounted for a 37.4% variance of the improvement in the contralateral bradykinesia–rigidity score on stimulation. This effect was frequency-specific, as this relationship was not observed with the low-*β* frequency band.

### Pathophysiology of low-*β* and high-*β* oscillations in the STN

The therapeutic outcome of DBS in PD is sought to be related to the modulation of the pathological oscillations in the STN area (Godinho et al., 2006; Accolla et al., 2016; Milosevic et al., 2020; Tamir et al., 2020; Kehnemouyi et al., 2021). Exaggerated synchronization in the *β* frequency range (13–35 Hz) is considered as an important biomarker of Parkinson's disease and linked to motor impairment (Brown, 2003; Hammond et al., 2007; Eusebio et al., 2009; Little and Brown, 2014). The suppression of *β* activities was evident with stimulation (Bronte-Stewart et al., 2009; Eusebio et al., 2011) and correlates with motor improvement (Kehnemouyi et al., 2021). Although studies have suggested different pathophysiological mechanisms and clinical relevance for the two *β* bands (Priori et al., 2004; Marceglia et al., 2006; Oswal et al., 2016; Godinho et al., 2021; Neuville et al., 2021), it remains unclear whether low-*β* (13–20 Hz) or high-*β* (20–35 Hz) oscillations recorded from the STN area in patients with PD would correlate with the DBS efficacy differently. Previous reports have suggested that low-*β* activity is suppressed with levodopa (Priori et al., 2004; Marceglia et al., 2006; Little et al., 2013), correlates with baseline symptoms (Neumann et al., 2016; van Wijk et al., 2016; West et al., 2016), relates to movement slowing (Lofredi et al., 2019), and is considered to be more directly pathological. One recent report demonstrated that low-*β* oscillation power was higher at the clinically chosen stimulation contacts than the non-active ones, suggesting that the therapeutic outcome of DBS might be through the modulation of the pathological low-*β* oscillations (Horn et al., 2017a).

However, it has also been proposed that stimulation at the surrounding tracts in the STN region is involved in the improvement of parkinsonian symptoms, particularly the hyperdirect pathway. The high-*β* oscillations were found to reflect the synchrony between the STN and the cortex and may indicate the hyperdirect pathway (Hirschmann et al., 2011; Litvak et al., 2011; Whitmer et al., 2012; van Wijk et al., 2016; Tinkhauser et al., 2018b). The cortical–STN coupling in the high-*β* range was reduced by DBS (Whitmer et al., 2012), and selective stimulation at the hyperdirect pathway effectively ameliorated parkinsonian symptoms (Gradinaru et al., 2009; Li et al., 2012; Miocinovic et al., 2018).

Though exaggerated low-*β* activities in the STN area were considered pathological and relevant to motor impairment in PD (Hammond et al., 2007), recent computational modeling studies suggested that the pathological synchrony in the low-*β* frequencies was induced by the hyperdirect pathway (Holgado et al., 2010; Pavlides et al., 2015; Oswal et al., 2021), an important functional connection between the motor cortex and the STN. Furthermore, previous work also demonstrated that high-*β* activities were an electrophysiological biomarker of the hyperdirect pathway (Oswal et al., 2016). Therefore, modulation of the high-*β* oscillations by DBS is likely to suppress the generation of pathological low-*β* oscillations and lead to the improvement of motor impairment in PD.

The significant association between the maximum high-*β* power and the improvement in bradykinesia–rigidity due to DBS in our study was consistent with the previous works using microelectrode recordings (Zaidel et al., 2010) and using LFP with a machine learning approach (Hirschmann et al., 2022). Both results suggested the high-*β* activities recorded in the STN region predicted the therapeutic benefit of DBS.

### The maximum high-*β* power correlated with the outcome of DBS of the STN

The significant correlation between high-*β* power at the STN and clinical improvement might have important clinical implications. The sampling of the maximum high-*β* power was at least partially relevant to the surgical trajectory of DBS lead implantation, given that the amplitude and power of LFP are likely to be affected by the deviation of the electrode from the oscillatory generator even for only a few millimeters (Chen et al., 2006b; Zaidel et al., 2010; Telkes et al., 2016). Therefore, accurate placement of DBS electrode in the STN region with greater high-*β* power may lead to a favorable outcome for DBS. However, high-*β* oscillations may also be related to the pathophysiology of PD. In particular, patients' demographic features, such as age, disease duration, the occurrence of dyskinesia, baseline motor impairment, and preoperative levodopa responsiveness, have been reported to be associated with DBS outcomes for PD (Charles et al., 2002; Welter et al., 2002; Jaggi et al., 2004; Pahwa et al., 2005; Kleiner-Fisman et al., 2006). We accordingly tested the relationship between these clinical variables and the oscillatory low-*β* and high-*β* powers, but no significant association was found.

The correlation between high-*β* power and improvement in bradykinesia–rigidity might be driven by the correlation between high-*β* and baseline motor impairment because patients with severe motor impairments tended to benefit from DBS more (Chen et al., 2006a; Schuepbach et al., 2019). We tested this hypothesis by correlating the motor scores off-stimulation with the two *β* subbands. However, no significant correlation was observed. This result is consistent with that of a recent study demonstrating that high-*β* oscillations in the STN particularly predicted the reduction of bradykinesia–rigidity due to stimulation but were not relevant to the patient's motor symptoms off-stimulation (Hirschmann et al., 2022).

However, it is important to stress that the possible relevance between the subband *β* activities and the clinical features still could not be excluded entirely. Studies using different approaches to analyze LFPs in the STN region have shown a direct correlation between the parkinsonian symptoms and the *β* stability index (Little et al., 2012), the *β* burst duration (Neuville et al., 2021), or the spatially extended coherence (Pogosyan et al., 2010) in the high-*β* range. It remains to be explored whether other more sophisticated approaches, which take into account the nonlinear relationship of LFP signals, can further identify the association between the *β* subbands activities and the clinical features of PD.

On the contrary to our data that demonstrated a significant positive correlation between high-*β* power in the STN and improvement in bradykinesia–rigidity due to DBS, a previous study reported a negative correlation between the *β* band oscillations and the therapeutic outcome of DBS (Ray et al., 2008). These divergent results might be explained by the different approaches employed in the two studies. Ray and colleagues measured the chronic DBS efficacy by contrasting the preoperative and postoperative motor scores. In contrast, the improvement in motor symptoms in our study was assessed when stimulation was switched on and off after surgery. Therefore, the DBS efficacy in our study could be more directly attributed to the stimulation itself and the potential confounding factors, such as disease progression or interaction with medication adjustment, could be avoided.

Another factor that may affect the direction of correlation is the “stun effect” (Chen et al., 2006b) in which pathological signals in the STN is temporarily disrupted during the penetration of the DBS electrode, and patients' symptoms improved even without stimulation. It has been argued that the better-localized electrodes might result in more disruption of *β* oscillations and ensure the benefits of STN stimulation. In our group, we routinely descended the DBS electrode slowly in steps of 1 mm during the electrode implantation to minimize the stun effect, allowing more residual pathological oscillatory activity to be recorded. Ultimately, by recording STN LFP from patients with chronically implanted bidirectional devices that allow sustained LFP data retrieval after surgery (Cummins et al., 2021), it might be possible to disentangle the confound of a stun effect on the correlation between the *β* power in the STN and the improvement in bradykinesia–rigidity.

The association between LFP power and clinical outcome was not only frequency-specific but also symptom-specific. No significant correlation was observed between the improvements in tremor and the low-*β* or high-*β* power in the present study. This is in line with the previous evidence showing that *β* oscillations were only correlated with bradykinesia–rigidity, but not tremor (Ray et al., 2008; Kühn et al., 2009; Zaidel et al., 2010). These observations imply that the pathology of tremors might involve different networks (Helmich et al., 2012). However, we did see a trend, although not significant, toward a negative correlation between the low-*β* power and the improvement in tremor. Recent studies have also shown that tremor is associated with the reduction of *β* oscillations (Qasim et al., 2016; Asch et al., 2020). Further study that takes into account the relationships between the oscillatory activities in the *β* and other frequency bands might provide more insights into the efficacy of DBS on tremors. The lack of association between *β* oscillations and improvement in tremor may also be explained by the ceiling effect of DBS efficacy for tremor, as the tremor was completely suppressed by stimulation in more than half of our patient cohort (20 out of 39 sides).

### DBS efficacy was correlated with the distance between the contact with the maximum high-*β* and that used for chronic stimulation

In addition to the accuracy of electrode implantation, the selection of the active contact may affect the therapeutic outcome. Previous studies suggest that stimulation at the DBS contact closest to the site with the maximum *β* power in the STN produces the greatest improvement in parkinsonian symptoms (Ince et al., 2010; Yoshida et al., 2010; Tinkhauser et al., 2018a; Milosevic et al., 2020). In the current study, we demonstrated that the distance between the depth of active contact and the maximum high-*β* was negatively correlated with the improvement in bradykinesia–rigidity in response to DBS. The greatest improvement was observed when the depth of active contact coincided with that of maximum high-*β* power. These results may help to identify the optimal therapeutic target in the STN area for DBS. Note that though this correlation was specific to the high-*β* frequency and not seen in low-*β* frequency, no significant difference was found between the depths of maximum low-*β* and high-*β* in the present study. It remains unclear whether the spatial distribution of low-*β* and high-*β* activities is identical in the STN area or not. One report suggested that high-*β* oscillations lie dorsally to low-*β* oscillations (Miyagi et al., 2009), but this has not been confirmed by later studies using multi-unit recording or the probabilistic LFP mapping method (Zaidel et al., 2010; Horn et al., 2017a). This ambiguity may be circumvented by intraoperative LFP recordings, which allow neuronal activities in the STN area to be recorded in 1-mm or smaller descending steps using the DBS electrode during electrode implantation; as such, the spatial resolution will be higher than the fixed bipolar recordings (Chen et al., 2006b).

The utility of an LFP-based approach to guide DBS programming is supported by a recent study combining multiple LFP spectral features to inform the selection of stimulation contact in PD (Shah et al., 2022). Compared with using *β*-range oscillations as the single feature, adding LFP features recorded from resting or movement states improved the accuracy of predicting the most satisfactory stimulating contact. This algorithm was particularly helpful for programming with multicontact directional DBS leads. In contrast to our results indicating that high-*β* activities in the STN were a good indicator for the outcome of DBS, Shah et al. reported that low-*β* activities correlated with clinical efficacy. However, they limited the clinical assessment only to upper-limb rigidity, whereas we examined tremor, axial symptoms, and combined rigidity and bradykinesia in the upper and lower limbs. STN activities in high-*β* were more connected to the motor cortex and considerably disrupted motor function. Modulation of high-*β* oscillations is likely to be related to different symptoms, such as bradykinesia–rigidity in four limbs (Little et al., 2012) or bradykinesia in the lower limbs (Tinkhauser et al., 2019). We determined the correlation of electrophysiological features with various parkinsonian symptoms, and the results might have crucial clinical applications to inform more personalized treatment strategies for patients with PD.

### High-*β* power and proximity of the contact for stimulation to the contact with maximum high-*β* inform the DBS efficacy

We identified at least two nonredundant, if not complementary, factors that were associated with the outcome of DBS therapy: the maximum high-*β* power and the distance between the contact with chronic stimulation and that of the maximum high-*β* activities. We combined these factors in a multiple regression model, as this method has been used in several electrophysiological studies to improve the predictive value (Ozkurt et al., 2011; Little et al., 2012; Kehnemouyi et al., 2021). We demonstrated that the combination of these factors predicted ~37.4% of the variation of improvement in the contralateral bradykinesia–rigidity scores in response to DBS. These results might inform both the therapeutic potential of an implanted electrode and the optimal active contact for chronic stimulation. This model may be used to predict more accurately the improvements in bradykinesia–rigidity of individual patients in response to DBS in the STN. In addition, our findings are also applicable to the development of closed-loop DBS. In contrast to the conventional DBS that suppressed broad spectral activities in the STN, the closed-loop DBS is more selective in its suppression of LFP. It remains to be seen whether the closed-loop DBS that is selectively triggered by the high-*β* oscillations is more effective than that triggered by the broad-band *β* oscillations.

The results of our hypothesis-driven study are consistent with those of a recent study that used machine learning techniques to analyze multiple features of LFP. Hirschmann et al. demonstrated that local high-*β* power was among the most crucial local features to predict DBS outcomes (Hirschmann et al., 2022). The prediction was not driven by symptom severity. Despite differences in methods and scope, the two studies reached the same conclusion that high-*β* power in the STN estimated improvement in bradykinesia–rigidity.

In contrast to their finding that the prediction of the DBS outcome was not due to the distance to the anatomical “sweet spot” in their study, we observed disparities in the depth selected for chronic stimulation and that the depth of the maximum high-*β*, which were purely based on electrophysiology rather than on imaging reconstruction, had a significant effect on DBS efficacy. Therefore, the present study indicated that high-*β* oscillations recorded from the DBS electrode can help evaluate the optimal stimulation contact for use in chronic stimulation.

### Limitations

There were several limitations in our study. First, one limitation inherent in this study is that the postoperative MR reconstruction was only available in five patients. It was not possible to compare the predicting value of electrophysiology to anatomy. Future work that correlated distance between anatomical sweet spot deriving from electrode reconstruction and stimulating contact with the clinical outcome will help to answer this question. This limitation is due to the fact that preoperative high-resolution MRI was only available in five of the patients. High-resolution MRI and tissue activation modeling approaches have suggested that stimulating the dorsolateral, sensorimotor STN produced the best outcome of DBS (Dembek et al., 2019; Kehnemouyi et al., 2021). Though the postoperative MRI attested to the accuracy of the electrode placement in our study, the locations of the contacts were presumptive and have not been confirmed by postoperative MR reconstruction. We elected to use an electrophysiological approach based on the DBS macroelectrode LFP recordings to test how well the high-*β* oscillations could predict the DBS efficacy, which proved to be valid.

Second, the present study only identified high-*β* oscillations as a single feature to estimate the therapeutic outcome. Combining multiple electrophysiological features and imaging markers resulted in higher prediction accuracy than that obtained by using beta activity alone (Hirschmann et al., 2022; Shah et al., 2022). However, the use of the dynamic high-*β* oscillations as a single feature is more intuitive and computation saving and can be a rational target for closed-loop DBS. The real-time rapid estimation of the LFP amplitude to deliver stimulation and the reduction of consumed electric energy are crucial for the closed-loop DBS regime.

Third, normalization by total power can be misleading if a strong tremor peak at 5 Hz exists. However, only 7 of the 26 patients in this study had tremor-dominant PD. A tremor in patients often subsided during LFP recording due to the stun effect occurring a few days after electrode implantation. Moreover, if a strong tremor peak causes a medium beta peak to appear small in the normalized version, it would lead to the underestimation of significance between beta oscillations and clinical improvement.

It should also be noted that our results came from a dataset with a limited number of patients recorded in the same center. Whether the result is generalizable requires further investigation.

## Conclusion

The high-*β* oscillations were associated with the bradykinesia–rigidity improvement from subthalamic DBS for PD patients. Both the maximum high-*β* activity recorded by the DBS macroelectrode and the proximity of the contact of stimulation to the contact with the max high-*β* were significantly correlated with stimulation efficacy. Our findings are important in informing the electrode implantation and selecting the optimal stimulation contact for chronic DBS. Combing these two factors may provide a more accurate estimation of a patient's response to DBS therapy.

## Data availability statement

The raw data supporting the conclusions of this article will be made available by the authors upon request, without undue reservation.

## Ethics statement

The studies involving human participants were reviewed and approved by Institutional Review Board and Ethics Committee of Chang Gung Memorial Hospital: CGMH-IRB No. 97-2345B. The patients/participants provided their written informed consent to participate in this study.

## Author contributions

C-CC gave the conception and established the team. Y-CC, P-LC, C-SL, and C-CC interviewed the patients and collected the data. P-LC and Y-CC organized the database. P-LC, H-TW, M-CC, and T-CL performed the signal analysis and the statistical analysis. P-HT and M-CY operated on the patients. C-HY managed brain images. P-LC and C-CC wrote sections of the manuscript. All authors contributed to manuscript revision, read, and approved the submitted version.

## Funding

This work was supported by the Ministry of Science and Technology, Taiwan (MOST108-2314-B-182-014-MY3 and MOST111-2321-B-A49-002), National Health Research Institutes, Taiwan (NHRI-EX111-11104NI), and the Chang Gung Memorial Hospital, Taiwan (CMRPG3B1432).

## Conflict of interest

The authors declare that the research was conducted in the absence of any commercial or financial relationships that could be construed as a potential conflict of interest.

## Publisher's note

All claims expressed in this article are solely those of the authors and do not necessarily represent those of their affiliated organizations, or those of the publisher, the editors and the reviewers. Any product that may be evaluated in this article, or claim that may be made by its manufacturer, is not guaranteed or endorsed by the publisher.
